# Hyperfocus or flow? Attentional strengths in autism spectrum disorder

**DOI:** 10.3389/fpsyt.2022.886692

**Published:** 2022-09-16

**Authors:** Annie Dupuis, Piyumi Mudiyanselage, Christie L. Burton, Paul D. Arnold, Jennifer Crosbie, Russell J. Schachar

**Affiliations:** ^1^Dalla Lana School of Public Health, University of Toronto, Toronto, ON, Canada; ^2^Neurosciences and Mental Health, Hospital for Sick Children, Toronto, ON, Canada; ^3^Department of Psychiatry, University of Toronto, Toronto, ON, Canada; ^4^The Mathison Centre for Mental Health Research and Education, Calgary, AB, Canada; ^5^Hotchkiss Brain Centre, University of Calgary, Calgary, AB, Canada

**Keywords:** autism spectrum disorder (ASD), attention-deficit/hyperactivity disorder (ADHD), impairment, attention, strengths, hyperfocus, flow

## Abstract

The comorbidity of autism spectrum disorder (ASD) and attention-deficit/hyperactivity disorder (ADHD) diagnoses is well established. An ASD diagnosis is associated with elevated ADHD traits and symptoms, as well as strengths in attention. In the ASD literature, attentional strengths have been described as maladaptive (e.g., hyperfocus), in contrast with positive portrayals in the typically developing population (e.g., flow). The objective of this study was to (1) compare profiles of attentional strengths and weaknesses in ASD and ADHD and (2) determine whether attentional strengths in ASD are associated with impairment, poorer cognitive flexibility, and perseveration/perfectionism. In a community sample of 5,744 children and youth, 131 children were reported as having a diagnosis of ASD (mean age 10.3 years) and 346 children were reported as having a diagnosis of ADHD (mean age 10.7 years). We used the Strengths and Weaknesses of Attention-Deficit/Hyperactivity-symptoms and Normal-behaviors (SWAN) rating scale to calculate attentional and hyperactive/impulse control strength and weakness counts and scores. The Autism-Spectrum Quotient Switching factor served as a measure of cognitive flexibility. Impairment was assessed with the Columbia Impairment Scale. We used the symmetry/ordering factor on the Toronto Obsessive-Compulsive Scale as a measure of perseveration/perfectionism. No differences were found between the ADHD and ASD groups in SWAN weakness scores, symptoms, or hyperactive/impulse control strengths; however, autistic children had higher rates of attentional strengths [odds ratio: 5.7, 95% CI (2.8, 11.6), *p* < 0.0001]. *Post-hoc* pairwise testing identified four attentional strengths with significantly higher rates in ASD than in ADHD. Attentional strength scores were not associated with impairment or poor cognitive flexibility, but predicted levels of perseveration/perfectionism. The effect of attentional strengths on impairment and cognitive flexibility did not differ between autistic and Control children, but the higher perseveration/perfectionism scores seen in ASD were not found in Control children. ASD is associated with a pattern of attentional strengths that is not found in ADHD Characterizing the full range of attentional abilities in autistic children may explain variability in outcomes such as quality-of-life indicators and identify protective factors, providing targets for strength-based behavioral interventions. The clinical and etiological implications of the subgroup of autistic children with attentional strengths require further investigation.

## Introduction

Autism spectrum disorder^1^ (ASD) is a neurodevelopmental disorder characterized by persistent deficits in social communication and social interactions and the presence of restricted, repetitive patterns of behaviors, interests, or activities (2). Elevated attention-deficit/hyperactivity disorder (ADHD) traits are common in ASD, with estimates ranging from 20 to 80% even in the absence of a comorbid ADHD diagnosis (3, 4). A recent meta-analysis of 89 studies published between 1993–2019 reported a pooled estimate of comorbid ADHD in ASD of 28% [95% CI (25,32)] (5), comparable to a meta-analysis of 18 studies in adults on the autism spectrum published between 2006 and 2016 where the pooled prevalence of comorbid ADHD was estimated to be 25.7% [95% CI (18.6, 34.3)] (6). Autistic individuals are also known to have the ability to remain intensely focused for prolonged periods. Fifty years ago, Lovaas et al. (7) coined the term “stimulus overselectivity” to describe patterns of focused attention seen in autistic children, although today the term “hyperfocus” is more commonly used. Within the ADHD and ASD literature, hyperfocus is often described through a negative lens. Lovaas et al. (7) claimed stimulus overselectivity was “excessive and maladaptive”. Isomura et al. (8) described attentional strengths as “excessive” and used the term “locked-in” when referring to an autistic person's focus on a subject of interest due to the resulting difficulty commanding their attention. In their review of the literature on cognitive flexibility in ASD, Geurts et al. (9) mentioned hyperfocus strictly in reference to its associated “difficulties in shifting attention”, and when summarizing descriptions of hyperfocus, Ozel-Kizil et al. (10) reported primarily negative language, describing it as “locking on to a task” in association with “difficulty of shifting their attention”, “neglect(ing) things”, and an inability to “give up what they are doing”. Hupfeld et al. (11) identified a perseverative/perfectionist dimension of hyperfocus, “getting ‘stuck on' small details,” following reports in open-ended interviews of adults with ADHD of “trying to get something just right” and “wanting everything to be perfect”.

In contrast to this negative perspective on over-focused attention in ASD and ADHD, the state of “flow” described in typically developing people is described similarly as intense absorption to the point of losing track of time and perception of the outside world but without the negative connotation associated with hyperfocus. The term “flow” was coined by Csikszentmihalyi (12) in reference to the “automatic, effortless, yet highly focused state” their study subjects described as the motivation for pursuing hobbies that were often challenging or even risky. While evidence that flow can facilitate greater achievement remains inconclusive, Schutte and Malouff (13) report that higher self-reported states of flow predict higher creativity scores on a novel task.

Ashinoff and Abu-Akel (14) proposed that hyperfocus and flow refer to the same concept, given their similarities, and identified four characteristic features: task engagement, heightened attention, diminished awareness of the environment, and improved performance. However, in a study of adults with ADHD, Hupfeld et al. (11) reported low to moderate correlations between scores on the Adult Hyperfocus Questionnaire (AHQ) and the LONG Dispositional Flow Scale-2-General (DFS-2). Given that most items on the flow questionnaire represent shallow flow states, they posited that flow varies from shallow flow to hyperfocus, a less common deep flow state. A more recent study among university students with and without ADHD symptoms (15) found negative correlations between scores across the subscales of the AHQ and the goals, feedback, concentration, and control subscales of the DFS-2, suggesting that the deep levels of absorption seen in hyperfocus are accompanied by perceptions of loss of control (i.e., the sense that one can accomplish anything), one of the nine dimensions of flow according to Csikszentmihalyi (16), and therefore may be impairing.

With the exception of one inattentive item, “often does not seem to listen when spoken to directly”, the DSM 5 does not explicitly include behaviors that could be construed as evidence of hyperfocus in its diagnostic criteria for ADHD (2). To find features that could reflect hyperfocus one would have to look at the *opposite* extreme of the high, symptomatic end of continuous ADHD traits (e.g., “sustain attention on tasks or play activities,” “ignore extraneous stimuli,” and “engage in tasks that require sustained mental effort”). Typical ADHD rating scales cannot identify strengths because they are limited to questions about the presence or absence of individual symptoms or deficits. Informants can rate a person as “not having a symptom” but cannot rate them as having an apparent strength in that trait (e.g., able to concentrate for prolonged periods of time). The development of the Strengths and Weaknesses of Attention-Deficit/Hyperactivity-symptoms and Normal-behaviors [SWAN (17)] has opened the door to conducting research across the full range of attentional abilities. Greven et al. (18) showed that the SWAN was associated with both positive (e.g., Subjective Happiness Scale, Life Satisfaction Scale) and negative (e.g., Mood and Feelings Questionnaire) outcomes across its full range of scores. Greven et al. (19) also found that low ADHD trait scores on the SWAN, representing apparent strengths, were associated with fewer internalizing and externalizing behavior problems and with better performance on visuospatial working memory, verbal working memory, and visual pattern recognition. In a large population study, Crosbie et al. (20) found that low ADHD trait scores predicted better stop signal reaction time, faster response time, and reduced response time variability on the stop signal task. Research to determine if apparent strengths in attention in autistic children are associated with positive outcomes as seen in typically developing children, and/or with impairment, difficulties with shifting their attention (cognitive flexibility), and perseveration/perfectionism as seen in hyperfocus is key to guiding treatment.

In this study, we use the SWAN questionnaire to identify attentional strengths and weaknesses in a large population sample of children and youth with a community diagnosis of ASD or ADHD. We hypothesized that the ASD group would be comparable to the ADHD group in the number of ADHD symptoms and weakness scores given the well-established and high rate of comorbid ADHD and subthreshold ADHD traits but would report higher rates of attentional strengths. In order to determine if attentional strengths are indicative of hyperfocus, we examined their association with measures of cognitive flexibility, impairment, and perseverative/perfectionist behaviors. In an exploratory objective, we looked at associations between attentional strengths and other ASD characteristics as reported on the remaining AQ-short-C factors and other OCD traits on the remaining TOCS factors to determine if attentional strengths are more common among those with specific ASD profiles or patterns of OC behaviors. Given that there is a trade-off between attentional strengths and weaknesses in that the more strengths one has the fewer weaknesses or symptoms one can have, we tested whether strengths and weaknesses make a unique, independent, contribution on each outcome. We estimated the effects of attentional strengths and weaknesses in a combined sample of Control and autistic children to determine if both ends of the attentional trait distribution function similarly between those with and without a diagnosis of ASD.

## Materials and methods

### Participants

Participants were drawn from the Spit for Science sample which included 5,743 participants (ages 4.0–19.0 years) recruited from a public science museum from 2019 to 2020 (see Supplemental materials for description of Spit 2 study and consort diagram). The current study is an extension of the 2009–2010 Spit for Science study, described in Crosbie et al. (20). A parent or youth self report of a diagnosis or treatment for ASD (*n* = 131, mean age = 10.3 years, 19.8% female) or ADHD (*n* = 346, mean age = 10.7 years, 33.5% female) by a health care practitioner was used to classify participants as meeting a community diagnosis for either disorder. Forty-five of the 131 autistic participants (34%) also had a diagnosis of ADHD. Participants with a diagnosis of ASD (*n* = 131) comprised 2.5% of the sample, and those with ADHD (*n* = 391) made up 7.4% of the sample (Supplementary Figure 1). Participants without a diagnosis of ASD or ADHD or elevated ADHD traits [SWAN total, inattentive, or hyperactive t-score > [98%ile] were classified as Controls (*n* = 4,726, mean age = 9.6 years, 52.3% females). Parents provided information about their child's diagnoses and behavior. Where parents were unavailable and youth were 12 or older, information was provided by self-report (ASD *n* = 11, ADHD *n* = 57). Given the large number of pairwise comparisons needed to compare the rates of strengths in ASD and ADHD across all SWAN items, we validated the results using an independent Spit for Science sample collected at the same location from 2009 to 2010 (20). The 2009–2010 sample (*n* = 16,675) included 224 children with a community diagnosis of ASD (mean age = 11.2 years, 14.3% female) and 968 children with a community diagnosis of ADHD (mean age = 11.6 years, 28.0% female). The AQ-short-C and the CIS questionnaires were not collected in the 2009–2010 Spit for Science study.

#### Measurement

##### Strengths and Weaknesses of Attention-Deficit/Hyperactivity-symptoms and Normal-behaviors

**Figure 1:**
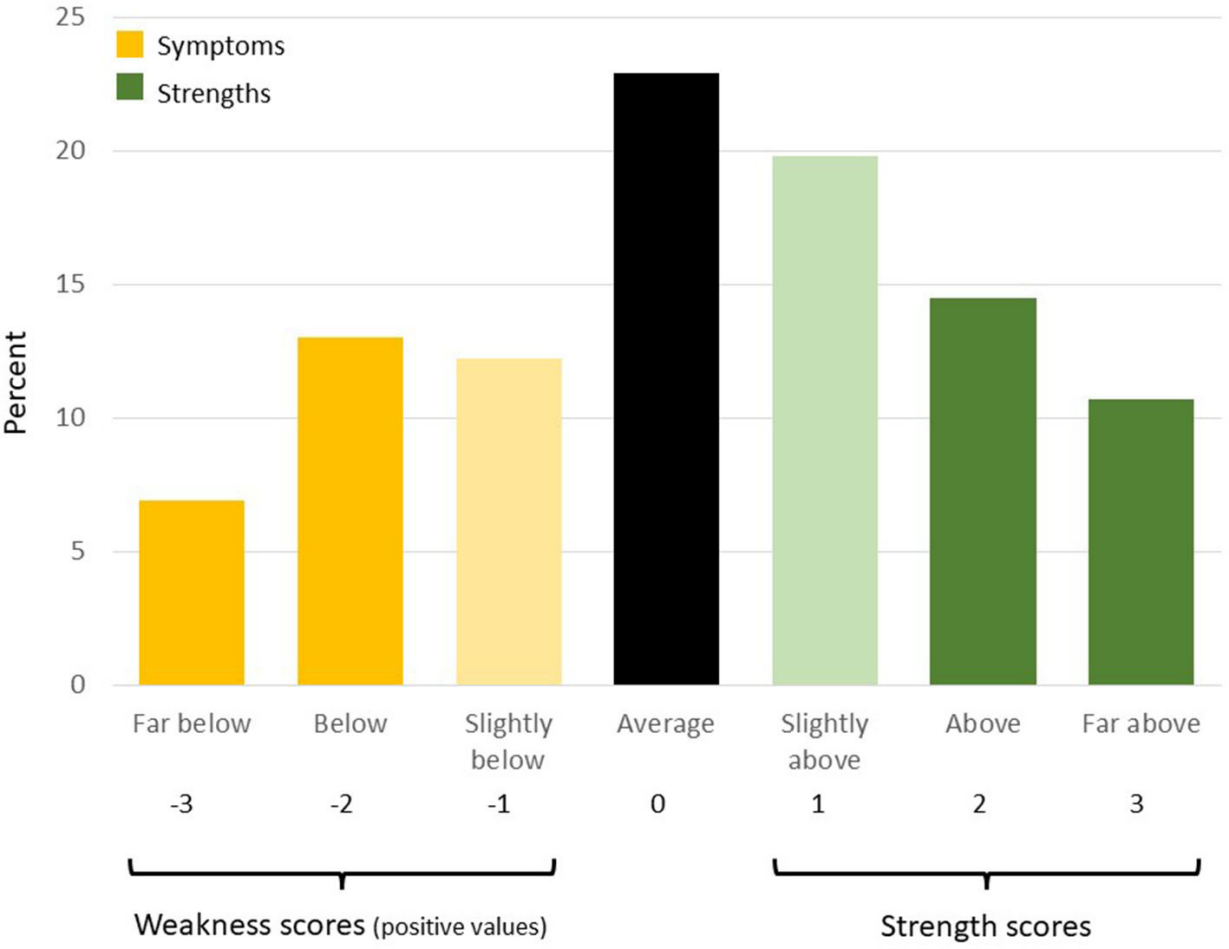
Strengths and weaknesses from SWAN scale. Creation of strength and weakness scores, symptoms and strengths, illustrated using the distribution (%) of responses to the item “Engage in tasks that require sustained mental effort” in the ASD sample. Scores that do not fall within the regions shown as “strength scores” or “weakness scores” are set to 0. We take the absolute value of weaknesses when calculating weakness scores so that slopes in the linear regression models retain a consistent meaning across both strength and weakness scores (positive slope represents increase in dependent outcome with more strengths or more weaknesses).

The SWAN (17, 21) consists of 18 items that mirror the DSM-5 ADHD symptoms. Each item is worded to describe a strength (e.g., attentional strength: “remembers daily activities”; hyperactive/impulse control strength: “sit still”) rather than a symptom, and items are rated on a 7-point scale ranging from −3 (far below average) to +3 (far above average) to assess both strengths and weaknesses within the previous 6 months. Using item response theory, the SWAN was found to be reliable across the full range of ADHD traits (22). Negative scores on the SWAN are considered weaknesses (ADHD traits) and positive scores are considered strengths. In order to compare the ASD and ADHD groups using scores comparable to those from ADHD screening tools that do not measure strengths, we created **weakness scores** by taking the absolute value of weakness ratings and assigning a score of 0 to scores reported as strengths (Figure 1). ADHD **symptoms** were estimated by treating weaknesses with a score of −2 or −3 as symptoms. Symptom counts are the total number of symptoms reported and range from 0 to 9 within each of the inattentive and the hyperactive/impulsive domains and 0–18 across all the SWAN items. We also describe apparent strengths using the same approach used for weaknesses by using scores above 0 as **strength scores** and assigning a value of 0 to scores reported as weaknesses, and **strengths** by treating scores of 2 or 3 as a strength. To characterize the full range of ADHD traits across both strengths and weaknesses, we report age, respondent, and gender standardized **t-scores** (23) across inattentive items, hyperactive/impulsive items, and all items (SWAN total) where higher t-scores correspond to higher ADHD traits. SWAN t-scores used to exclude high ADHD trait from the Control sample did not control for gender. The use of the SWAN has been validated across both parent respondents and youth respondents (24).

##### Autism quotient (AQ-short-C)

We used an abridged version of the AQ–child (25), selecting the same 28 items as those from the AQ-short for adults (26). Respondents were asked to think about behaviors over the previous 6 months. Items on the AQ-short-C ask about both high and low ASD trait levels (e.g., “They find it hard to make new friends,” “They enjoy meeting new people”) except items from the Numbers and Patterns factor which only ask about behaviors associated with high ASD traits. Items also measure high and low trait levels by providing both positive and negative response options, with four response options ranging from “strongly disagree” to “strongly agree”. To maintain consistency with item scoring for other outcome measures and facilitate comparisons of slopes across different models, items were scored at −3, −1, 1, and 3 for the four response options from the lowest ASD trait level to the highest ASD trait level. Items were grouped using the five factors identified in a factor analysis by Hoekstra et al. (26): Switching, Social Skills, Routine, Numbers and Patterns, and Imagination. One item, “new situations make them anxious”, which loaded on different factors in the English and Dutch samples studied by Hoekstra, was included in the “Routine” factor where it fit more closely in our data. The AQ-short-C Switching factor consists of items that measure difficulties with cognitive flexibility that may be indicative of hyperfocus (e.g., “they frequently get so strongly absorbed in one thing that they lose sight of other things” and “If there is an interruption, they can switch back to what they were doing very quickly”).

##### Columbia impairment scale

The Columbia Impairment Scale (27) consists of thirteen items used to assess impairment across different settings and activities. To maintain a consistent metric with other outcomes and facilitate comparisons of slopes across the different models, the 5 response choices were scored as −3, −1.5, 0, 1.5, and 3 for responses ranging from “not a problem” (−3) to “very bad problem” (3). For consistency with the other measures (SWAN, AQ-Short-C, and TOCS), raters were asked to think about the last 6 months. Both parent and youth forms of the CIS have been validated (20).

##### Toronto obsessive compulsive scale

Each of the 28 items on the TOCS (28) is worded to describe an OCD thought or behavior (e.g., “repeat actions before they seem quite right”). Like the SWAN, items are rated on a 7-point scale ranging from far below (−3) to far above average (+3) to capture both extremes of the OCD trait continuum within the previous 6 months. Factor analysis identified 6 factors: Cleaning/Contamination, Symmetry/Ordering, Counting/Checking, Rumination, Superstition, and Hoarding (29). The symmetry/ordering domain in OCD has been linked to perfectionism (24) and may capture perseverative behaviors similar to those found in the “Getting ‘stuck on' small details” dimension of the Adult Hyperfocus Questionnaire (11), e.g., “worries a lot if something is not exactly the way he/she likes”, “repeat actions before they seem quite right”. The TOCS has been validated across both parent and youth respondents (26).

Where questionnaire items were missing, pro-rated totals were used (*n* = 2 Control, *n* = 2 ASD, and *n* = 0 ADHD participants were missing 1 SWAN item; *n* = 7 Controls were missing one AQ item and *n* = 3 Controls were missing 2 AQ items; *n* = 8 Controls were missing one TOCS item and *n* = 6 Controls were missing 2 TOCS items). Missing items were treated as missing in strength prevalence figures.

### Ethical considerations

Participation starts with a discussion of the study's purpose followed by informed consent from parents and verbal assent from children or informed consent from youth, which has been approved by The Hospital for Sick Children's research ethics board. Research staff are trained to assess competence to consent. Data is anonymized prior to genetic analysis. Some participants leave before finishing all aspects of the study typically because of time constraints.

### Statistical analyses

We first tested our hypothesis that the ASD and ADHD groups would appear similar when compared using weakness scores and symptom counts as defined in Figure 1 using independent sample *t*-tests for weakness scores and Wilcoxon Rank Sum tests for symptom counts. Second, we used independent sample *t*-tests to compare the ASD and ADHD groups using SWAN t-scores that are based on both strengths and weaknesses. Then, we tested our hypothesis that autistic children would have more reported individual SWAN strengths than children with ADHD using a repeated measures logistic regression model, with the presence/absence of a strength on each item as our dependent variable and controlling for age and/or gender where significant. We then included an item x group interaction term in each model and conducted pairwise comparisons to identify specific items whose rates differ between the ASD and ADHD groups using a *post-hoc* Tukey Adjustment for multiple comparisons. Given the large number of pairwise comparisons this entailed, we validated the results by running the same analyses in the Spit for Science 2009–2010 sample.

To assess whether attentional strengths are associated with impairment, difficulties with cognitive flexibility, and perseveration/perfectionism, we first looked at the univariable association between attentional strength scores with the following dependent variables: Columbia Impairment Scale as a measure of impairment, the AQ-short-C Switching factor as a measure of cognitive flexibility, and the TOCS symmetry/ordering factor as a measure of perseveration/perfectionism. Attentional strength scores are inversely correlated (r = −0.47) with attentional weakness scores since every item that is reported as a strength is an item that cannot be reported as a weakness. Outcomes that were significantly associated with attentional strengths in univariable models were therefore also analyzed in multivariable linear regression models that also controlled for attentional weakness scores, as well as age and gender where significant. Controlling for attentional strengths and attentional weaknesses together in the same model allowed us to test whether strength scores have an independent effect on the outcome over and above their shared variance with attentional weakness scores. In a sensitivity analysis, we also ran the multivariable models in the ASD sample with no comorbid diagnosis of ADHD and in the sample with parent respondents only. The same approach was used to examine the association between attentional strengths and ASD traits, as measured by each of the remaining AQ-short-C factors in separate models, and OC traits as measured by each of the remaining TOCS factors. Finally, to compare the effect of attentional strengths between autistic and Control children, we reran the multivariable models in a combined ASD and Control sample and compared the effect of attentional strengths between the two groups by testing an interaction term between group and attentional strength scores.

## Results

When we compared the ADHD and ASD samples using SWAN weakness scores (“high ADHD”, i.e., scores where strengths are given a score of 0) and using symptom counts, there were no significant differences between the two groups (Table 1). However, when we compared the two groups using SWAN total t-scores based on both strengths and weaknesses, autistic children had significantly lower inattention t-scores (i.e., less inattentive) than children with ADHD (P = 0.0009), but not hyperactive/impulsive t-scores (*p* = 0.52, Table 1), suggesting that differences between the two groups on overall SWAN t-scores are driven primarily by differences in apparent attentional strengths not assessed by weakness scores or symptom counts. We also report SWAN weakness scores, symptom counts, and total t-scores among ASD participants with and without a comorbid ADHD diagnosis (Supplementary Table 2).

**Table 1:** Comparison of SWAN weakness, symptom, and total t-scores between the ADHD and ASD groups.

	**ASD**	**ADHD**	**p**
**Mean SWAN weakness (ADHD) scores**^a^ **(sd)**			
SWAN total	1.0 (0.7)	1.1 (0.7)	0.48
SWAN inattentive	1.0 (0.8)	1.1 (0.8)	0.15
SWAN hyperactive	1.0 (0.7)	1.0 (0.8)	0.83
**Median SWAN symptoms**^b^ **(IQR)**^c^			
SWAN total	4 (2, 10)	5 (2, 10)	0.47
SWAN inattentive	2 (0, 6)	3 (0, 6)	0.15
SWAN hyperactive	2 (1, 5)	2 (0, 5)	0.73
**Mean SWAN t-scores**^d^ **(sd)**			
SWAN total	61.6 (10.1)	64.0 (9.6)	0.021
SWAN inattentive	59.6 (11.0)	63.3 (9.8)	0.0009
SWAN hyperactive	62.2 (9.8)	62.9 (10.1)	0.52

SWAN symptom counts: Wilcoxon Rank Sum test; SWAN weakness scores, SWAN t-scores: *t*-test.

^a^SWAN weakness scores are the absolute value of average item scores where strengths have all been set to a score of 0. Scores calculated in this way are similar to scores obtained from ADHD tools that truncate scores at 0 in the absence of symptoms.

^b^SWAN symptoms are defined as weaknesses with a score of −2 or −3. Inattentive and hyperactive/impulsive symptom counts have a range of 0–9 and total symptom counts have a range of 0–18.

^c^IQR, Interquartile Range.

^d^SWAN *t*-scores are based on SWAN total across all strengths and weaknesses, and control for gender and age.

### Prevalence of ADHD strengths

**Figure 2:**
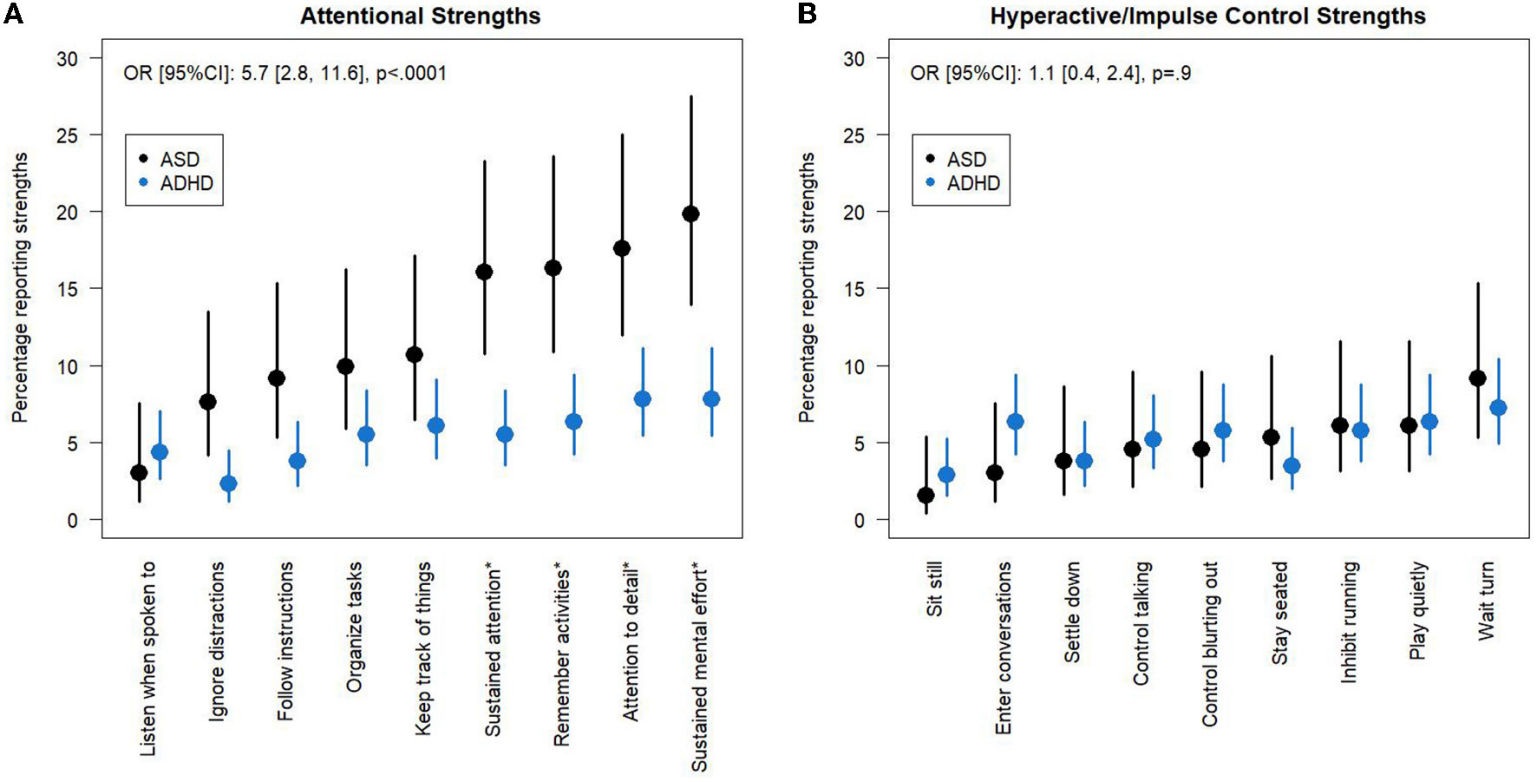
Attentional and hyperactive/impulse control strengths in ADHD and ASD groups. Percentage (95% CI) of ASD and ADHD groups who report **(A)** attentional strengths and **(B)** hyperactive/impulse control strengths. Items with a score of 2 or 3 are classified as strengths. Odds Ratios were estimated for the group effect in models with no group x item interaction term. Items that are significantly different between the two groups using a *post-hoc* Tukey Adjustment in a model with a group x interaction term are indicated by a * in the axis label. CI, Confidence Interval.

Across all items, autistic children reported attentional strengths significantly more frequently than children with ADHD [Odds Ratio: 5.7, 95% CI: (2.8, 11.6), *p* < 0.0001, Figure 2; Supplementary Table 3]. There were no significant differences in the prevalence of hyperactive/impulse control strengths between the two groups (ASD prevalence 4.9%, ADHD prevalence 5.2%, *p* = 0.9). We then included an item x group interaction (χ^2^ = 12.1, *p* = 0.15) to the attentional strengths model and, using a *post-hoc* Tukey adjustment for multiple comparisons, identified four attentional strengths that were significantly more common in ASD than ADHD: “sustain attention on tasks or play activity,” ”engage in tasks that require sustained mental effort,” “remember daily activities,” and “give close attention to detail/avoid careless mistakes.” We also compared the prevalence of strengths between the ASD and ADHD groups in the 2009/10 Spit for Science sample to validate our results and the same four attentional strengths were reported significantly more often in ASD than ADHD with no differences in hyperactive/impulse control strengths (Supplementary Figure 2).

### Cognitive flexibility, impairment, and perseveration/perfectionism

Higher attentional strength scores in children on the autism spectrum were associated with significantly better cognitive flexibility as measured in the AQ-short-C [β = −0.52, 95% CI: (−0.91, −0.13), *p* = 0.010] and reduced impairment as measured using the Columbia Impairment Scale [β = −0.41, 95% CI: (−0.77, −0.05), *p* = 0.025] in univariable models (Table 2). However, greater attentional strength scores predicted greater perseveration/perfectionism as measured by the TOCS symmetry/ordering factor [β = 0.60, 95% CI: (0.27, 0.93), *p* = 0.0005, Table 2].

**Table 2:** Effect of SWAN attentional strength scores and SWAN attentional weakness scores in the ASD sample, univariable and multivariable models^a^.

**Outcomes^b^ (dependent variables)**	**SWAN attentional strength score**		**SWAN attentional weakness score**	
**Univariable models**				
	β **[95% CI**^c^**]**	***p***	β **[95% CI**^c^**]**	***p***
**CIS** (impairment)	−0.41 [−0.77, −0.05]	0.025	0.51 [0.29, 0.73]	<0.0001
**TOCS** symmetry/ordering	0.60 [0.27, 0.93]	0.0005	−0.25 [−0.86, −0.31]	0.029
**AQ-short-C** switching	−0.52 [−0.91, −0.13]	0.010	0.57 [0.33, 0.82]	<0.0001
Patterns and numbers	0.90 [0.39, 1.42]	0.0007	−0.14 [−0.50, 0.21]	0.43
	**Multivariable models**			
**CIS** (impairment)	0.00 [−0.40, 0.40]	0.91	0.51 [0.25, 0.78]	0.0002
**TOCS** symmetry/ordering	0.55 [0.16, 0.95]	0.006	−0.06 [−0.32, 0.20]	0.81
**AQ-short-C** switching	−0.09 [−0.53, 0.35]	0.70	0.54 [0.25, 0.84]	0.0003
Patterns and numbers	1.05 [0.47, 1.64]	0.0005	0.20 [−0.19, 0.59]	0.31

^a^Univariable models control for attentional strengths score or attentional weakness score only. Multivariable models control for both attentional strength and weakness scores as well as gender, where significant. Age was not a significant predictor in any of the multivariable models. Gender was significant in the AQ-short-C Numbers and Patterns model. Females reported average AQ-short-C Numbers and Patterns scores that were 1.00 lower than males (95% CI: [−1.63, −0.38], *p* = 0.002). Cells with non-significant effects are shaded in.

^b^Model outcomes are the average item score for each domain to maintain a consistent metric across domains based on different numbers of items. TOCS items have 7 response options with scores ranging from −3 to 3. AQ-short-C has four response options scored as −3, −1, 1, and 3 and the Columbia Impairment Scale (CIS) has 5 response options that are assigned values of −3, −1.5, 0, 1.5, and 3 so that item averages for each outcome have a comparable potential range of values.

^c^CI, Confidence Interval.

Only outcomes with significant univariable attentional strength effects are shown; see Supplementary Table 4 for all univariable attentional strength effects.

Attentional weakness scores were also significant predictors of cognitive flexibility, impairment, and perseveration/perfectionism in univariable models (Table 2). There was a moderate inverse correlation between attentional strength and attentional weakness scores (r = −0.47). We checked to see if higher levels of attentional strengths were associated with better cognitive flexibility or reduced impairment given the same attentional weakness score. In the multivariable models of average AQ-short-C switching item scores, attentional weakness scores were statistically significant [β = 0.54, 95% CI: (0.25, 0.84), *p* = 0.0003] but not attentional strengths (*p* = 0.70, Table 2). Similarly, higher attentional weakness scores predicted greater average impairment scores [β = 0.51, 95% CI: (0.25, 0.78), *p* = 0.0002], eliminating the univariable effect of attentional strengths (*p* = 0.91, Table 2). Thus, given the same attentional weakness score, higher levels of attentional strengths were not associated with better cognitive flexibility or reduced impairment. In multivariable analysis, the TOCS symmetry/ordering factor was significantly associated with attentional strengths [β = 0.55, 95% CI: (0.16, 0.95), *p* = 0.006] after controlling for attentional weakness scores, which were no longer significant (*p* = 0.81, Table 2). That is, the effect of attentional traits on the TOCS symmetry/ordering factor was driven by the presence of strengths and not of weaknesses.

**Figure 3:**
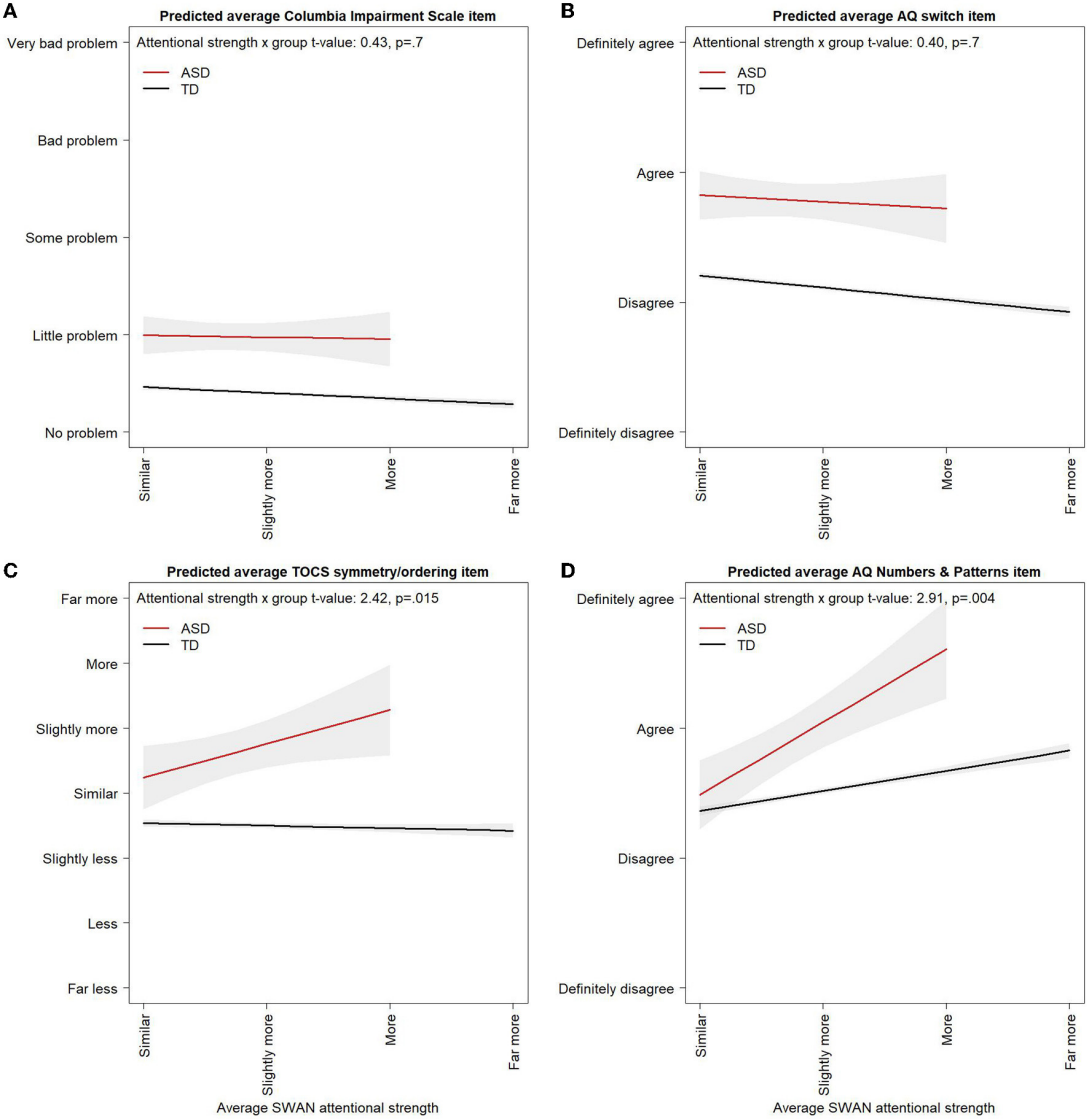
Correlates of attentional strengths in ASD and controls. Multivariable linear regression model predicted values for **(A)** average AQ switching item, **(B)** average Columbia Impairment Scale item, **(C)** average TOCS symmetry/ordering item, and **(D)** average AQ-short-C Numbers and Patterns item across average attentional strength scores in the ASD and Control samples. Models control for age, gender, and attentional weakness scores. Multivariable linear regression model predicted values are shown for 11-year-old males with attentional weakness scores of 0. Varying the age or weakness score at which predicted values from the linear regression model are obtained would raise or lower the lines but would not alter the slopes or the difference between the ASD and Control groups. Predicted values for the ASD group are shown up to a maximum attentional strength score of 2 in orders to avoid extrapolating outside the observed range of the data. Some AQ-short-C items are reverse coded so that “definitely agree” always corresponds to the highest ASD trait level across all items. 95% Confidence Intervals are shown in light gray.

The effect of attentional strength scores on cognitive flexibility and impairment was not significantly different in the ASD and the Control samples (AQ-short-C switching model: SWAN attentional strength score x group interaction *p* = 0.6; Columbia Impairment Scale model: SWAN attentional strength score x group interaction *p* = 0.6, Figure 3). No effect of attentional strengths was found on the TOCS symmetry/ordering factor in the Control sample [Control attentional strengths effect: −0.01, 95% CI: (−0.06, 0.04), difference in effect from attentional strength effect in ASD *p* = 0.016, Figure 3].

There was evidence of a floor effect on the Columbia Impairment Scale. With the five item response options scored from −3 to 3, the predicted average item score from the multivariable linear regression model at an attentional weakness score of 0 in the ASD group was −1.53 [95% CI: (−1.73, −1.32)] corresponding to a response of “very little problem” and, in the Control group, was −2.41 [95% CI: (−2.44, −2.39)], just above a response of “no problem” providing little room for improvement with increasing attentional strength scores (Figure 3).

There was no evidence that higher scores on the TOCS symmetry/ordering factor in ASD was driven by individuals with a comorbid diagnosis of OCD. Only two autistic children reported a comorbid OCD diagnosis: one child had an attentional strength score of 0.8 and the second child had an attentional strength score of 0, and neither reported OCD symptoms in the symmetry/ordering domain. Although TOCS symmetry/ordering scores increased significantly with higher attentional strength scores, the average TOCS symmetry/ordering item predicted from the multivariable linear regression model across the range of observed attentional strength scores all fell below the cut-off for an OCD symptom.

### ASD and OCD traits

In a univariable model, higher levels of attentional strengths were significantly associated with higher average AQ-short-C numbers and patterns scores [β = 0.90, 95% CI: (0.39, 1.42), *p* = 0.0007, Table 2]. The association remained after controlling for attentional weakness scores, which were not significant in either a univariable or multivariable model, and gender [Female effect: −1.00, 95% CI: (−1.63, −0.38), *p* = 0.002, Table 2]. Attentional strengths were also associated with higher scores on the AQ-short-C numbers and patterns factor in a multivariable model including Control individuals, but the effect was significantly smaller in Controls than in ASD [Control attentional strengths effect: 0.31, 95% CI: (0.26, 0.36), difference in effect from attentional strength effect in ASD *p* = 0.005, Figure 3]. Attentional strengths did not show any significant univariable associations with the AQ-short-C social, routine, or imagination factors or the TOCS cleaning/contamination, counting/checking, rumination, superstition, or hoarding factors (Supplementary Table 4).

In a sensitivity analysis, we repeated all the multivariable models in the ASD–ADHD sample only and in the parent respondent sample (Supplementary Table 5). Results did not vary from those across the entire ASD sample.

## Discussion

The goal of this study was to characterize attentional strengths in ASD. We took advantage of the unique structure of the SWAN ADHD questionnaire to create measures of ADHD weaknesses, ADHD strengths, ADHD symptoms and overall total ADHD scores. The ASD group had comparable levels of ADHD symptoms and ADHD weaknesses as the ADHD group consistent with the literature showing high rates of comorbid ADHD and high subthreshold ADHD traits among ASD individuals. However, when we expanded the focus to the full range of ADHD trait scores measuring both strengths and weaknesses, we discovered that children on the autism spectrum had significantly lower total scores than those with ADHD. These differences in trait profiles were driven by an increase in apparent attentional strengths in autistic children. Four attentional strengths were significantly more common in ASD than in ADHD: “sustain attention on tasks or play activity,” “engage in tasks that require sustained mental effort,” “remember daily activities,” and “give close attention to detail/avoid careless mistakes.” ADHD and ASD did not differ in the proportion of hyperactive/impulsive control strengths. These results were replicated in an independent sample, revealing a consistent profile of apparent attentional strengths in ASD across different samples.

The four apparent attentional strengths identified as being more common in ASD than in ADHD are well established ASD traits. Both the Weak Central Theory of autism (30) and the hyper-systemizing theory (31) presume excellent attention to detail, and a high prevalence of strengths in sustained attention and tasks that require sustained mental effort is consistent with the patterns of focused attention identified by Lovaas et al. (7). While the literature on memory in ASD is dominated by studies of impaired or unimpaired performance on cognitive tasks with few reports of superior performance (32, 33), Kanner's original description (34) of “autistic” behaviors in children describes one child as having “an unusual memory for faces and names” and another child with “an exceptional rote memory”. In semi-structured interviews of 28 autistic adults to identify ASD traits that have had a positive impact on their lives, focus and attention-to-detail were the most commonly reported strengths, followed by memory, retention of facts, organization, and creativity (35). Even a cursory internet search using the key words “autism” and “strengths” returns numerous lists of strengths in autism, all of which include “focus,” “attention to detail,” and “memory.” Our study is the first to use the SWAN to quantify the prevalence of these strengths in a large community sample of children and youth with autism spectrum.

In much the same way that hyperfocus describes attentional strengths through a negative lens and flow through a positive lens, the Weak Central Theory of autism describes a preference for local processing (attention to detail) stemming from difficulties understanding the big picture, in contrast with the hyper-systemizing theory where strengths in attention to detail are an asset used in support of recognizing patterns and rules that govern systems (36). Given these opposing perspectives on attentional strengths and to better understand how they impact autistic children, we tested the association between attentional strength scores with impairment, cognitive flexibility, and perseveration/perfectionism. We found that greater attentional strengths are associated with better cognitive flexibility and lower impairment. However, these associations were related to the fact that more strengths necessarily imply fewer weaknesses. Once the association between strengths and weaknesses was taken into account, attentional strengths were not associated with cognitive flexibility and impairment. The association between attentional strengths and impairment did not differ between ASD and Control individuals suggesting a unified conceptualization of these strengths across these groups (rather than negative hyperfocus vs. positive flow). Of note, the absence of an advantageous effect of attentional strengths on impairment likely reflects a floor effect of the measure given that the Columbia Impairment Scale is a global measure of impairment that assesses difficulties across multiple settings and activities but does not measure strengths in functioning in the absence of problems.

While there is no doubt that attentional strengths can be seen as beneficial in ASD, as one autistic adult explains, “I believe the unwavering focus to a subject has aided me academically” (35), autistic adults report both advantages and problems, linking attention to detail with difficulty switching tasks and perfectionism. Although we did not find any evidence that attentional strengths are associated with more difficulty switching tasks as measured using the AQ-short-C switching factor, we found a significant association between attentional strength scores and perseveration/perfectionism as measured by the symmetry/ordering factor on the TOCS. We also found a significant association between attentional strength scores and the AQ-short-C numbers and patterns factor in both the ASD and Control samples. Four of the five items included in the numbers and patterns factor on the AQ-short-C are found in the attention to detail factor in the original AQ (37). While factor analyses of the AQ have yielded inconsistent results, confirmatory factor analysis of 11 models (38) shows strong support for factor solutions that group attention to detail items with items relating to fascination with numbers and patterns, supporting our finding of an association between the two domains across the Control and ASD samples.

The attentional strengths identified as more common in ASD than ADHD are well established ASD behaviors, yet research on comorbid ASD and ADHD has focused on weaknesses that are common to both disorders and not on the apparent attentional strengths that may serve to discriminate between them. More research is needed to characterize the co-occurrence of apparent strengths and weaknesses to inform ADHD diagnostic decisions in individuals on the autism spectrum. The ability to enter a state of intense focus depends on task engagement and does not preclude the presence of difficulties with focus in other contexts. That is, the same individual may sometimes exhibit attentional strengths and at other times attentional weaknesses on the same SWAN item, depending on their interest in and enjoyment of their current activity. This apparent contradiction may underlie some cases of poor respondent agreement on ADHD questionnaires. In their expert consensus guidelines, Young et al. (39) warn that “concentration problems and/or overactivity may be less evident when individuals are engrossed in a topic or activity of interest”. Much the same way that a child can become fully absorbed when engaged but also experience impairing levels of inattention otherwise, a child with an exceptional rote memory (e.g., memory for movie/television dialogue) may still consistently forget to hand in their homework or bring their sneakers home for the weekend. Until profiles that include both apparent strengths and weaknesses are better understood, the presence of attentional strengths on a screening tool should not be used to rule out an ADHD diagnosis, but rather, further information should be obtained in a follow-up interview to ensure that strengths are not masking weaknesses.

Our findings highlight the importance of designing questionnaires that measure both extremes of a behavioral trait. Such questionnaires can improve the homogeneity of empirically defined phenotypic subgroups, increasing our power to identify susceptibility genes in linkage studies (40) as well as in genome wide association studies (41). In their study reporting on the results of confirmatory factor analyses of 11 published models of the AQ, English et al. (38) found that the attention to detail factor did not correlate with the other factors in the optimal solution. When conducting cluster analyses to identify homogeneous subgroups, variables that are highly correlated tend to produce clusters that differ in degree and not in type. In contrast, when one or more variables are not correlated with the others, cluster analysis can result in subgroups that show marked differences in their profiles. This was the case with a cluster analysis conducted by Kitazoe et al. (42) using the original AQ factors: two factors were identified with high AQ scores, one of which included those with high scores across all the factors with the other having high scores on all but the attention to detail factor. Our results provide further evidence that elevated scores on the AQ-short-C numbers and patterns factor, concurrent with attentional strengths, is not the norm in ASD but rather, identifies a subgroup with a distinct profile. In a population-based study, Happé et al. (43) found that elevated scores on items measuring repetitive behaviors, and in particular, noticing and remembering details, were the greatest predictors of high levels of talent in math, music, art, or memory. In a large study of university students, Billington et al. (44) found that elevated systemizing scores predicted entry into the physical sciences at university. Further research is needed to replicate these results in a sample of individuals on the autism spectrum. A longitudinal study of autistic children that have attentional strengths and high systemizing scores can provide invaluable information on their long-term trajectories and outcomes.

Hyperfocus or flow? Our failure to find a significant association between attentional strength scores with impairment and cognitive flexibility in either Control or autistic children belies the conventional belief that high levels of focused attention in Control children is a strength, as represented by flow, but invariably maladaptive in autistic children. Whether this is because some children on the autism spectrum experience high levels of strengths without the challenges associated with hyperfocus while others exclusively experience hyperfocus, or whether both positive and negative levels of attention can be seen within the same child remains to be determined. Compared to flow research, the research in hyperfocus is in its infancy. With more study, a consensus on the distinguishing features, overlap, and boundaries between flow and hyperfocus can be established, facilitating research into the conditions that lead to more positive flow experiences rather than negative hyperfocus ones.

## Limitations

We characterized a broad range of attentional strengths in autistic children as described by parents using a single score to describe the prevailing trend across each item in the previous 6-month period. As such, we were unable to determine if individuals can present with both strengths and weaknesses for the same item across different contexts. Individuals with high strength scores may present with different profiles of strengths, not all of which are characteristic of hyperfocus (e.g., “Listen when spoken to directly”). Participants visiting a science center may not be representative of or generalizable to the general population. Notably, there were fewer individuals from the lowest SES quintile and more high SES individuals participating in the 2009–2010 study than across the surrounding area (20). The study used parent or youth self reports of diagnosis and behavior which may be less accurate than a clinically ascertained sample. However, we note that the prevalence of ADHD and ASD and their comorbidities are similar to what is reported in studies using more thorough diagnostic assessments and that community diagnosis predicts executive functions and genetic risk factors similarly to clinically diagnosed samples (20). The AQ-C has been validated for youth respondents but the subset of items used in the AQ short has only been validated in adults. Although we did not find any significant effects of respondent in any of our models, the youth self-report sample was small and underpowered to detect respondent effects. There is a need for more studies across different populations using questionnaires specifically designed to assess both hyperfocus and flow, as well as positive outcome measures that may be associated with higher attentional strengths.

## Data availability statement

The raw data supporting the conclusions of this article will be made available by the authors, without undue reservation.

## Ethics statement

The studies involving human participants were reviewed and approved by Hospital for Sick Children Research Ethics Board. Written informed consent to participate in this study was provided by the participants' legal guardian/next of kin.

## Author contributions

AD and PM performed the statistical analyses. AD wrote the first draft of the manuscript. All authors contributed to the conception and design of the study, manuscript revision, read, and approved the submitted version.

## Funding

This work was supported by two Canadian Institutes of Health Research grants (PJT-159462 and PJT-165876), Susan Bradley Health Clinician Scientist Endowment (JC) and the Alberta Innovates Translational Health Chair in Child and Youth Mental Health (PA).

## Conflict of interest

The authors declare that the research was conducted in the absence of any commercial or financial relationships that could be construed as a potential conflict of interest.

## Publisher's note

All claims expressed in this article are solely those of the authors and do not necessarily represent those of their affiliated organizations, or those of the publisher, the editors and the reviewers. Any product that may be evaluated in this article, or claim that may be made by its manufacturer, is not guaranteed or endorsed by the publisher.

## Footnotes

^1^We recognize that language is important when writing about autism and that preferences vary within the autism community. Following recommendations by Botha et al. (1), we use both “autistic child” and “child on the autism spectrum” throughout when referring to autistic individuals and ASD when referring to diagnosis and as an abbreviation for the group label.

## References

[1] Botha M Hanlon J Williams GL. Does language matter? Identity-first versus person-first language use in autism research: a response to vivanti. J Autism Dev Disord. (2021) 1–9. 10.1007/s10803-020-04858-w [Epub ahead of print]. 33474662 PMC7817071

[2] APA. Diagnositic and Statistical Manual of Mental Disorders. 5th ed., Text Rev. Arlington, VA: American Psychiatric Publishing (2022).

[3] Matson JL Rieske RD Williams LW. The relationship between autism spectrum disorders and attention-deficit/hyperactivity disorder: an overview. Res Dev Disabil. (2013) 34:2475–84. 10.1016/j.ridd.2013.05.021 23751293

[4] Van Der Meer JMJ Oerlemans AM Van Steijn DJ Lappenschaar MGA De Sonneville LMJ Buitelaar JK. Are autism spectrum disorder and attention-deficit/hyperactivity disorder different manifestations of one overarching disorder? Cognitive and symptom evidence from a clinical and population-based sample. J Am Acad Child Adolesc Psychiatry. (2012) 51:1160–72. 10.1016/j.jaac.2012.08.024 23101742

[5] Lai M-C Kassee C Besney R Bonato S Hull L Mandy W. Prevalence of co-occurring mental health diagnoses in the autism population: a systematic review and meta-analysis. Lancet Psychiatry. (2019) 6:819–29. 10.1016/S2215-0366(19)30289-5 31447415

[6] Lugo-Marín J Magán-Maganto M Rivero-Santana A Cuellar-Pompa L Alviani M Jenaro-Rio C. Prevalence of psychiatric disorders in adults with autism spectrum disorder: a systematic review and meta-analysis. Res Autism Spectr Disord. (2019) 59:22–33. 10.1016/j.rasd.2018.12.004

[7] Lovaas OI Schreibman L Koegel R Rehm R. Selective responding by autistic children to multiple sensory input. J Abnorm Psychol. (1971) 77:211–22. 10.1037/h0031015 5556929

[8] Isomura T Ogawa S Shibasaki M Masataka N. Delayed disengagement of attention from snakes in children with autism. Front Psychol. (2015) 6:241. 10.3389/fpsyg.2015.00241 25784895 PMC4347301

[9] Geurts HM Corbett B Solomon M. The paradox of cognitive flexibility in autism. Trends Cogn Sci. (2009) 13:74–82. 10.1016/j.tics.2008.11.006 19138551 PMC5538880

[10] Ozel-Kizil ET Kokurcan A Aksoy UM Kanat BB Sakarya D Bastug G. Hyperfocusing as a dimension of adult attention deficit hyperactivity disorder. Res Dev Disabil. (2016) 59:351–8. 10.1016/j.ridd.2016.09.016 27681531

[11] Hupfeld KE Abagis TR Shah P. Living “in the zone”: hyperfocus in adult ADHD. Atten Defic Hyperact Disord. (2019) 11:191–208. 10.1007/s12402-018-0272-y 30267329

[12] Csikszentmihalyi M. Creativity: The Psychology of Discovery and Invention. New York, NY: Harper Collins Publishers Ltd. (2013).

[13] Schutte NS Malouff JM. Connections between curiosity, flow and creativity. Pers Individ Dif. (2020) 152:109555. 10.1016/j.paid.2019.109555

[14] Ashinoff BK Abu-Akel A. Hyperfocus: the forgotten frontier of attention. Psychol Res. (2021) 85:1–19. 10.1007/s00426-019-01245-8 31541305 PMC7851038

[15] Grotewiel MM Crenshaw ME Dorsey A Street E. Experiences of hyperfocus and flow in college students with and without Attention Deficit Hyperactivity Disorder (ADHD). Curr Psychol. (2021) 2022:1–11. 10.1007/s12144-021-02539-0

[16] Csikszentmihalyi M. Flow: The Psychology of the Optimal Experience. 2nd ed. New York, NY: Harper & Row (2002).

[17] Swanson JM Schuck S Porter MM Carlson C Hartman CA Sergeant JA. Categorical and dimensional definitions and evaluations of symptoms of ADHD: History of the SNAP and the SWAN rating scales. Int J Educ Psychol Assess. (2012) 10:51–70. 26504617 PMC4618695

[18] Greven CU Merwood A Van Der Meer JMJ Haworth CMA Rommelse N Buitelaar JK. The opposite end of the attention deficit hyperactivity disorder continuum: genetic and environmental aetiologies of extremely low ADHD traits. J Child Psychol Psychiatry. (2016) 57:523–31. 10.1111/jcpp.12475 26474816 PMC4789118

[19] Greven CU van der Meer JMJ Hartman CA Lappenschaar MGA Buitelaar JK Rommelse NNJ. Do high and low extremes of ADHD and ASD trait continua represent maladaptive behavioral and cognitive outcomes? A population-based study. J Atten Disord. (2018) 22:924–32. 10.1177/1087054715577136 25823744

[20] Crosbie J Arnold P Paterson A Swanson J Dupuis A Li X. Response inhibition and ADHD traits: correlates and heritability in a community sample. J Abnorm Child Psychol. (2013) 41:497–507. 10.1007/s10802-012-9693-9 23315233 PMC3600128

[21] Burton CL Wright L Shan J Xiao B Dupuis A Goodale T. SWAN scale for ADHD trait-based genetic research: a validity and polygenic risk study. J Child Psychol Psychiatry Allied Discip. (2019) 60:988–97. 10.1111/jcpp.13032 30908652

[22] Greven CU Buitelaar JK Salum GA. From positive psychology to psychopathology: the continuum of attention-deficit hyperactivity disorder. J Child Psychol Psychiatry. (2018) 59:203–12. 10.1111/jcpp.12786 28731214

[23] Schachar RJ Dupuis A Anagnostou E Georgiades S Soreni N Arnold PD. Obsessive-compulsive disorder in children and youth: neurocognitive function in clinic and community samples. J Child Psychol Psychiatry. (2022) 63:881–9. 10.1111/jcpp.13533 34687037

[24] Olatunji BO Ebesutani C Tolin DF. A bifactor model of obsessive beliefs: specificity in the prediction of obsessive-compulsive disorder symptoms. Psychol Assess. (2019) 31:210–25. 10.1037/pas0000660 30307265

[25] Auyeung B Baron-Cohen S Wheelwright S Allison C. The autism spectrum quotient: children's version (AQ-Child). J Autism Dev Disord. (2008) 38:1230–40. 10.1007/s10803-007-0504-z 18064550

[26] Hoekstra RA Vinkhuyzen AAE Wheelwright S Bartels M Boomsma DI Baron-Cohen S. The construction and validation of an abridged version of the autism-spectrum quotient (AQ-Short). J Autism Dev Disord. (2011) 41:589–96. 10.1007/s10803-010-1073-0 20697795 PMC3076581

[27] Bird HR Andrews H Schwab-Stone M Goodman S Dulcan M Richters J. Global measures of impairment for epidemiologic and clinical use with children and adolescents. Int J Methods Psychiatr Res. (1996) 6:295–307. 10.1002/(SICI)1234-988X(199612)6:4<295::AID-MPR173>3.3.CO;2-5

[28] Park LS Burton CL Dupuis A Shan J Storch EA Crosbie J. The Toronto obsessive-compulsive scale: psychometrics of a dimensional measure of obsessive-compulsive traits. J Am Acad Child Adolesc Psychiatry. (2016) 55:310–8.e4. 10.1016/j.jaac.2016.01.008 27015722

[29] Burton CL Park LS Corfield EC Forget-Dubois N Dupuis A Sinopoli VM. Heritability of obsessive-compulsive trait dimensions in youth from the general population. Transl Psychiatry. (2018) 8:191. 10.1038/s41398-018-0249-9 30228290 PMC6143601

[30] Frith U. Autism: Explaining the Enigma. Oxford, UK: Basil Blackwell (1989).

[31] Happé F. Autism. London: UCL Press (1996).

[32] Boucher J Mayes A Bigham S. Memory in autistic spectrum disorder. Psychol Bull. (2012) 138:458–96. 10.1037/a0026869 22409507

[33] Fan Siu NY Jiaying LE J. A review of the verbal memory profile of individuals with autism spectrum disorder. J Psychol Clin Psychiatry. (2014) 2:00054. 10.15406/jpcpy.2014.02.00054

[34] Kanner L. Autistic disturbance of affective contact. Nerv Child. (1943) 2:217–50.

[35] Russell G Kapp SK Elliott D Elphick C Gwernan-Jones R Owens C. Mapping the autistic advantage from the accounts of adults diagnosed with autism: a qualitative study. Autism Adulthood. (2019) 1:124–33. 10.1089/aut.2018.0035 31058260 PMC6493410

[36] Baron-Cohen S Ashwin E Ashwin C Tavassoli T Chakrabarti B. Talent in autism: hyper-systemizing, hyper-attention to detail and sensory hypersensitivity. Philos Trans R Soc Lond B Biol Sci. (2009) 364:1377–83. 10.1098/rstb.2008.0337 19528020 PMC2677592

[37] Baron-Cohen S Wheelwright S Skinner R Martin J Clubley E. The autism-spectrum quotient (AQ): evidence from Asperger syndrome/high-functioning autism, males and females, scientists and mathematicians. J Autism Dev Disord. (2001) 31:5–17. 10.1023/A:1005653411471 11439754

[38] English MCW Gignac GE Visser TAW Whitehouse AJO Maybery MT. A comprehensive psychometric analysis of autism-spectrum quotient factor models using two large samples: model recommendations and the influence of divergent traits on total-scale scores. Autism Res. (2020) 13:45–60. 10.1002/aur.2198 31464106

[39] Young S Hollingdale J Absoud M Bolton P Branney P Colley W. Guidance for identification and treatment of individuals with attention deficit/hyperactivity disorder and autism spectrum disorder based upon expert consensus. BMC Med. (2020) 18:146. 10.1186/s12916-020-01585-y 32448170 PMC7247165

[40] Shao Y Raiford KL Wolpert CM Cope HA Ravan SA Ashley-Koch AA. Phenotypic homogeneity provides increased support for linkage on chromosome 2 in autistic disorder. Am J Hum Genet. (2002) 70:1058–61. 10.1086/339765 11875756 PMC379103

[41] Manchia M Cullis J Turecki G Rouleau GA Uher R Alda M. The impact of phenotypic and genetic heterogeneity on results of genome wide association studies of complex diseases. PLoS One. (2013) 8:1–7. 10.1371/journal.pone.0076295 24146854 PMC3795757

[42] Kitazoe N Fujita N Izumoto Y Terada SI Hatakenaka Y. Whether the autism spectrum quotient consists of two different subgroups? Cluster analysis of the autism spectrum quotient in general population. Autism. (2017) 21:323–32. 10.1177/1362361316638787 27132011

[43] Happé F Vital P. What aspects of autism predispose to talent? Philos Trans R Soc Lond B Biol Sci. (2009) 364:1369–75. 10.1098/rstb.2008.0332 19528019 PMC2677590

[44] Billington J Baron-Cohen S Wheelwright S. Cognitive style predicts entry into physical sciences and humanities: questionnaire and performance tests of empathy and systemizing. Learn Individ Differ. (2007) 17:260–8. 10.1016/j.lindif.2007.02.004

