# Supplementary material for: Hyperfocus or flow? Attentional strengths in autism spectrum disorder
Source: Front Psychiatry. 2022 Sep 16;13:886692. doi: 10.3389/fpsyt.2022.886692 (PMC9579965; doi:10.3389/fpsyt.2022.886692)
Supplement: Supplementary file 1 [file Data_Sheet_1.pdf]

Supplemental Material

Spit for Science was conducted in collaboration with the Ontario Science Centre which is a public space for the discovery of science. Since 1969, more than 54 million people from all over Ontario, Canada, North America, and the world have attended. About 80% of visitors are from Ontario. About 1 million people visit each year of which about 165,000 are school children. Community access programmes assure that attendees from all walks of life can attend. **Research Live!** is a programme that allows visitors to take part in actual scientific research and to contribute to scientific progress during their visit. Many interactive exhibits are operated by Science Centre staff; others are operated by universities and research institutes in the region. Spit for Science is an exhibit within the interactive area of the Science Centre which exhibits facts and artifacts about genes and behavior (posters, etc). Visitors approach the exhibit to ask questions about it and are greeted by our hosts who answer their questions and invite them to participate in the study. Most participants come with a parent, but some older children come to the exhibit on their own. The Science Centre allows a 30-minute limit to participation in any project. Participation starts with a discussion of the study's purpose followed by informed consent which has been approved by the sponsoring institution, in our case The Hospital for Sick Children's research ethics board. Research staff are trained to assess competence to consent. Participation involves completion of rating scales to measure behavioral traits (see Methods), measurement of height and weight, completion of a brief computerized game that measures response inhibition and reaction time, and spitting into a small tube that allows us to extract DNA. Participants are rewarded with a small gift or gift certificate such as $5.00 toward movie tickets. No feedback about performance is provided and data is anonymized prior to genetic analysis. Some participants leave before finishing all aspects of the study typically because of time constraints. The project is ongoing.

Supplemental Figure 1: Consort diagram

n=5,743

↓ Consent withdrawn n=22 (0.4%)

n=5,721

↓ missing age n=2 (<0.1%) or gender n=201 (3.5%)

n=5,518

	n	ASD n	ADHD n	male n	mean age (sd)
No SWAN	233	4	5	116	9.5 (2.9)

↓

n=5,285

ASD +/- ADHD	ADHD +/- ASD
131 (2.5%)	391 (7.4%)

↓ High ADHD trait (SWAN t-scores > 70 (98%ile) but no ADHD or ASD diagnosis (n=82)

n=5,203

Sample used in summary statistics of SWAN variables. For model purposes, participants with comorbid ASD and ADHD are included in the ASD group:

	ASD	ADHD	Control
n	131	346	4,726
male n (%)	105 (80.2)	230 (66.5)	2,255 (47.7)
mean age (sd)	10.3 (3.0)	10.7 (2.9)	9.6 (3.0)

Missing values by model outcome:

	n	ASD n	male n	mean age (sd)
No TOCS	46	0	23	10.3 (3.0)
No AQ	129	0	73	10.5 (3.1)
No CIS	947	0	486	9.8 (2.8)

Supplemental Figure 2: Difference in percentage reporting (a) attentional strengths and (b) hyperactive/impulse control strengths[1] in the ASD and ADHD groups in the 2009/10 and 2019/20 Spit for Science samples.

a. Attentional strengths

b. Hyperactive/impulse control strengths

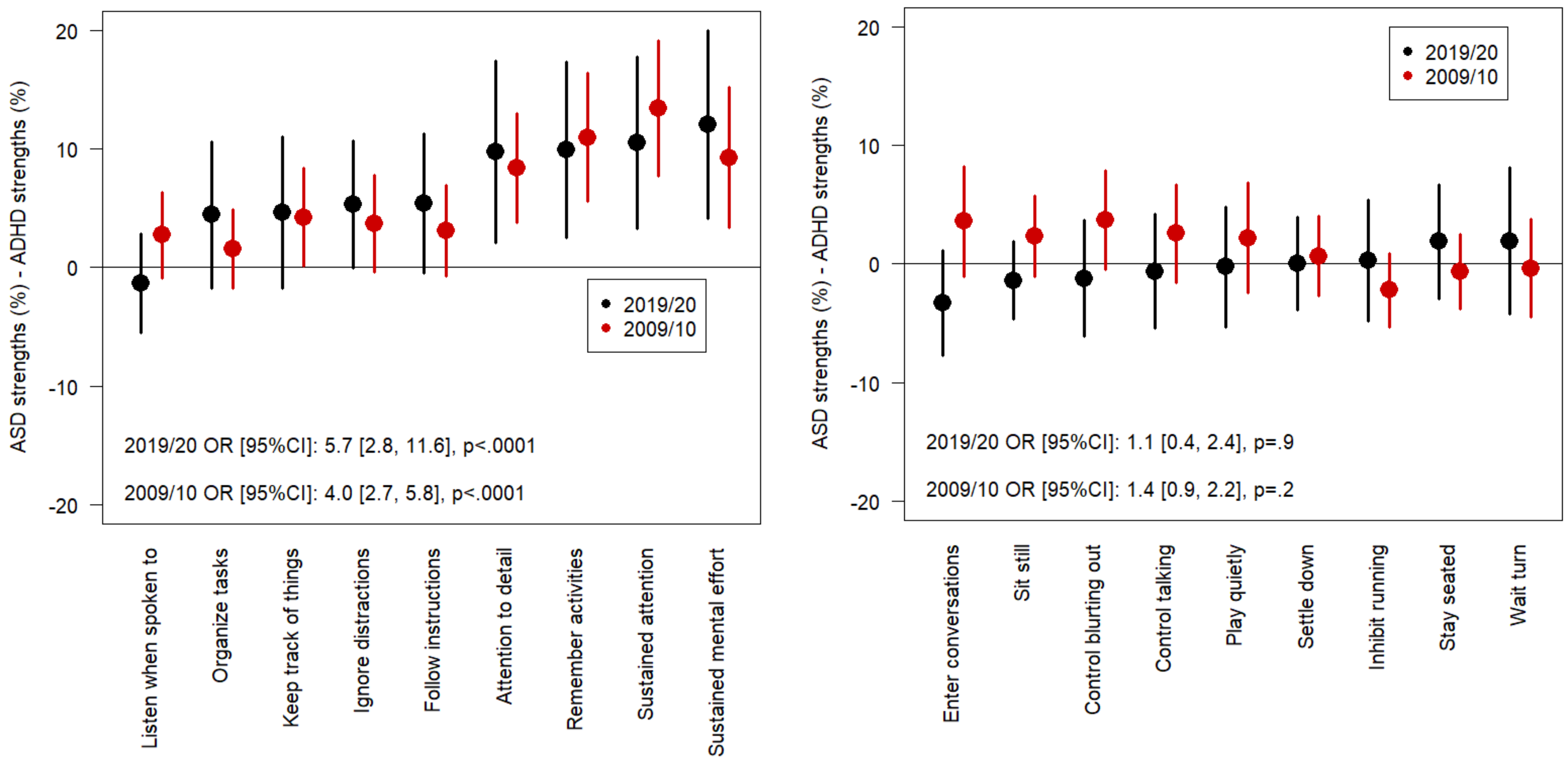

[1] Items with a score of 2 or 3 are classified as strengths.

Each point represents the difference in the percentage of strengths reported between the ASD and the ADHD groups. For example, in the 2019-2020 sample, 19.8% of autistic children report strengths on the item “Engage in tasks that require sustained mental effort” compared to only 7.8% of children with ADHD, for a difference of 12.0% between the two groups, represented by the last black point in the first panel. All but one point in the first panel are above 0, indicating that autistic children report more strengths than children with ADHD in both samples across all but one item, with the same 4 items showing differences that are significantly greater than 0 across both samples. In contrast, points representing the differences in hyperactive/impulsive control strengths hover around the 0 line with some points falling above and some below the line.

Supplemental Table 1: Derivation of independent and dependent questionnaire variables

SWAN variables	Items	Method	Range
SWAN item scoring	18	-3 Far Below (High ADHD trait)<br>-2 Below<br>-1 Slightly Below<br>0 Average<br>1 Slightly Above<br>2 Above<br>3 Far above (Low ADHD trait)	-3, 3
SWAN symptom count		SWAN items with scores of -2 or -3 are treated as	
Inattentive	9	symptoms. Symptom counts consist of the total	0, 9
Hyperactive/Impulsive	9	number of symptoms in a domain or across the	0, 9
Total	18	entire scale.	0, 18
SWAN strengths count		SWAN items with scores of 2 or 3 are treated as	
Inattentive	9	strengths. Strength counts across domains are not	0, 9
Hyperactive/Impulsive	9	reported. Rather, we report the prevalence of	0, 9
Total	18	strengths across each individual item.	0, 18
SWAN t-scores		The sum of SWAN items are standardized by	
Inattentive	9	respondent, age, and gender. T-scores have a	1, 89[1]
Hyperactive/Impulsive	9	mean of 50 and sd of 10, higher t-scores	1, 90
Total	18	correspond to higher ADHD traits.	1 – 97
SWAN weakness scores		The absolute value of the sum of negative SWAN	
Inattentive[2]	9	items divided by the total number of items in the	0 – 3
Hyperactive/Impulsive	9	domain (strengths contribute a value of "0" to the	0 – 3
Total	18	sum). Higher weakness scores correspond to<br>more weaknesses.	0 – 3
SWAN strength scores		The sum of SWAN items greater than 0 (ratings	
Inattentive[2]	9	above average) divided by the total number of	0 – 3
Hyperactive/Impulsive	9	items in the domain (weaknesses contribute a	0 – 3
Total	18	value of "0" to the sum).	0 – 3
**Dependent variables**	**Items**	**Method**	**Range**
**Columbia Impairment Scale**[3]	13	Average item score<br>-3 No problem<br>-1.5 Little problem<br>0 Some<br>1.5 Very bad problem<br>3 Bad problem	-3, 3
AQ-short-C		Average item score (Reverse-coded items)	-3, 3
Switching[3]	4	-3 Definitely Agree (Disagree)	
Social Skills	7	-1 Slightly Agree (Disagree)	
Routine	4	1 Slightly Disagree (Agree)	
Number and Patterns	5	3 Definitely Disagree (Agree)	
Imagination	8		
TOCS		Average item score	-3, 3
Symmetry/Ordering[3]	4	-3 Far less	
Cleaning/Contamination	5	-2 Less	

Counting/Checking	3	-1	Slightly less
Rumination	2	0	Similar to others
Superstition	3	1	Slightly more
Hoarding	2	2	More
		3	Far more

[1] No limits to possible values; range shown is the observed range in the sample

[2] SWAN inattentive weakness and strength scores are included as independent variables in univariable and multivariable models

[3] Columbia Impairment Scale, AQ-short-C Switching factor (cognitive flexibility), and TOCS symmetry/ordering factor (perfectionism/perseveration) are included as dependent variables (outcomes) in models testing the primary hypotheses.

Supplemental Table 2: Clinical characteristics of the ASD group by ADHD diagnosis.

**Mean SWAN weakness scores[1] (sd)**	**ASD + ADHD**	**ASD - ADHD**
SWAN total	1.3 (0.7)	0.8 (0.6)
SWAN inattentive	1.5 (0.7)	0.7 (0.7)
SWAN hyperactive	1.2 (0.7)	0.9 (0.7)
**Median SWAN symptoms[2] (IQR)**		
SWAN total	8 (3,12)	4 (1,7)
SWAN inattentive	5 (2,7)	1 (0,3)
SWAN hyperactive	3 (1,5)	2 (0,4)
**Mean SWAN t-scores[3] (sd)**		
SWAN total	67.1 (9.2)	58.9 (9.4)
SWAN inattentive	66.5 (9.3)	56.1 (10.2)
SWAN hyperactive	65.0 (9.1)	60.8 (9.8)

[1] SWAN weakness scores are the absolute value of average item scores where strengths have all been set to a score of 0. Scores calculated in this way are similar to scores obtained from ADHD tools that truncate scores in the absence of symptoms. [2] SWAN symptoms are defined as weaknesses with a score of -2 or -3. Inattentive and hyperactive/impulsive symptom scores have a range of 0-9 and total symptom scores have a range of 0-18. [3] SWAN t-scores are based on SWAN total scores across all strengths and weaknesses, and control for gender and age

Supplemental Table 3: Percentage [95% CI[2]] of individuals with a community diagnosis of ASD or ADHD as well as Control participants reporting strengths[1] in attention and hyperactive/impulse control

	ASD	ADHD	Control
**Attention**			
Listen when spoken to directly	3.1 [1.2,7.6]	4.3 [2.6,7.0]	29.6 [28.3,30.9]
Ignore extraneous stimuli	7.6 [4.2,13.5]	2.3 [1.2,4.5]	14.9 [14.0,16.0]
Follow through on instructions & finish school work/chores	9.2 [5.3,15.3]	3.8 [2.2,6.3]	31.4 [30.1,32.7]
Organize tasks and activities	9.9 [5.9,16.2]	5.5 [3.5,8.4]	25.9 [24.7,27.2]
Keep track of things necessary for activities	10.7 [6.5,17.1]	6.1 [4.0,9.1]	25.8 [24.7,27.2]
Sustain attention on tasks & play activities	16.0 [10.7,23.3]	5.5 [3.5,8.4]	31.7 [30.4,33.1]
Remember daily activities	16.3 [10.9, 23.6]	6.4 [4.2,9.4]	24.6 [23.4,25.8]
Give close attention to detail/avoid careless mistakes	17.6 [12.0,25.0]	7.8 [5.4,11.1]	26.9 [25.6,28.1]
Engage in tasks that require sustained mental effort	19.8 [13.9,27.5]	7.8 [5.4,11.1]	32.3 [31.0,33.6]
**Hyperactive/impulsive control**			
Sit still [control movements of hands/feet or control squirming]	1.5 [0.4,5.4]	2.9 [1.6,5.2]	22.1 [21.0,23.3]
Enter into conversations and games [control interrupting/ intruding]	3.1 [1.2,7.6]	6.4 [4.2,9.4]	23.5 [22.3,24.7]
Settle down and rest [control constant activity]	3.8 [1.6, 8.6]	3.8 [2.2,6.3]	23.9 [22.7,25.1]
Modulate verbal activity [Control excess talking]	4.6 [2.1 9.6]	5.2 [3.3,8.1]	20.9 [19.7,22.0]
Reflect on questions [control blurting out answers]	4.6 [2.1 9.6]	5.8 [3.8,8.8]	24.9 [23.7,26.2]
Stay seated [when required by class rules / social conventions]	5.3 [2.6,10.6]	3.5 [2.0,6.0]	30.2 [28.9,31.6]
Modulate motor activity [Inhibit in appropriate running / climbing]	6.1 [3.1, 11.6]	5.8 [3.8,8.8]	25.1 [23.9,26.4]
Play quietly [keep noise level reasonable]	6.1 [3.1, 11.6]	6.4 [4.2,9.4]	28.7 [27.4,30.0]
Await turn [stand in line and take turns]	9.2 [5.3, 15.3]	7.2 [4.9,10.4]	33.2 [31.8,34.5]

[1] Items with a score of 2 or 3 are classified as strengths. [2] CI: Confidence Interval

Supplemental Table 4: Association between SWAN attentional strength scores with the Columbia Impairment Scale, the TOCS Symmetry/Ordering factor, and the AQ-short-C Switching factor and other ASD and OCD trait domains as measured by the AQ-short-C and the TOCS.[1]

**Outcomes**	**SWAN attentional strength score**	
	β [95% CI[2]]	p
**CIS** Impairment	-0.41 [-0.77,-0.05]	.025
**TOCS** Symmetry/Ordering	0.60 [0.27,0.93]	.0005
**AQ-short-C** Switching	-0.52 [-0.91,-0.13]	.010
	β [95% CI[2]]	p
**AQ-short-C**		
Patterns/Numbers	0.90 [0.39,1.42]	.0007
Social	0.29 [-0.15,0.72]	.19
Routine	0.08 [-0.38,0.53]	.74
Imagination	-0.20 [-0.54,0.14]	.24
**TOCS**		
Cleaning/Contamination	-0.07 [-0.42,0.28]	.69
Counting/Checking	0.28 [-0.12,0.67]	.16
Rumination	-0.15 [-0.59,0.29]	.51
Superstition	-0.23 [-0.59,0.14]	.23
Hoarding	-0.04 [-0.48,0.40]	.86

[1] Model outcomes are the average item score for each domain to maintain a consistent metric across domains based on different numbers of items. TOCS items have 7 response options with scores ranging from -3 to 3. AQ-short-C items have four response options scored as -3, -1, 1, and 3 and the CIS has 5 response options that are assigned values of -3, -1.5, 0, 1.5, and 3 so that

item averages for each outcome have a comparable potential range of values. Cells where the association is not statistically significant have been shaded in. [2] CI: Confidence Interval

Supplemental Table 5: Effect of SWAN attentional strength scores and SWAN attentional weakness scores in the ASD - ADHD subsample (n=86) and the sample with parent respondents only (n=120), multivariable models[1].

	**Multivariable Models**			
**Outcomes**[2] (dependent variables)	**SWAN attentional strength score**		**SWAN attentional weakness score**	
**ASD – ADHD sample**	*β* [95% CI[3]]	*p*	*β* [95% CI[3]]	*p*
**CIS** Impairment	0.06 [-0.33,0.46]	.76	0.60 [0.28,0.93]	.0004
**TOCS** Symmetry/Ordering	0.59 [0.15,1.04]	.009	-0.08 [-0.45,0.28]	.65
**AQ-short-C** Switching	-0.02 [-0.47,0.42]	.90	0.74 [0.37,1.10]	.0002
Patterns & Numbers	0.95 [0.34,1.57]	.003	0.05 [-0.46, 0.56]	.84
**Parent respondents**	*β* [95% CI[3]]	*p*	*β* [95% CI[3]]	*p*
**CIS** Impairment	.03 [-0.42,0.47]	.91	0.56 [0.28,0.84]	.0001
**TOCS** Symmetry/Ordering	0.58 [0.16,1.01]	.008	0.00 [-0.27,0.27]	>.99
**AQ-short-C** Switching	0.15 [-0.29,0.59]	0.51	0.68 [0.40,0.96]	<.0001
Patterns & Numbers	1.03 [0.40,1.65]	.002	0.09 [-0.30,0.49]	0.64

[1] Multivariable models control for both attentional strength and weakness scores as well as gender, where significant. Age was not a significant predictor in any of the multivariable models. Gender was significant in the AQ-short-C Numbers and Patterns model. Females reported average AQ-short-C Numbers and Patterns scores that were 1.28 lower than males (95%CI: [-2.01,-0.56], p=.0007) in the ASD – ADHD model and 1.31 lower than males (95%CI: [-1.97,-0.64], p=.0002) in the Parent respondent model. Cells with non-significant effects are shaded in.

[2] Model outcomes are the average item score for each domain to maintain a consistent metric across domains based on different numbers of items. TOCS items have 7 response options with scores ranging from -3 to 3. AQ-short-C has four response options scored as -3, -1, 1, and 3 and the Columbia Impairment Scale has 5 response options that are assigned values of -3, -1.5, 0, 1.5,

and 3 so that item averages for each outcome have a comparable potential range of values. [3] CI: Confidence Interval
